# RP11‐81H3.2 promotes gastric cancer progression through miR‐339‐HNRNPA1 interaction network

**DOI:** 10.1002/cam4.2867

**Published:** 2020-02-13

**Authors:** Fen‐Rong Chen, Su‐Mei Sha, Shen‐Hao Wang, Hai‐Tao Shi, Lei Dong, Dong Liu, Yan Cheng, Miao An, Yan Wang, Jun Zhang

**Affiliations:** ^1^ Department of Gastroenterology The Second Affiliated Hospital of Xi'an Jiaotong University Xi’an China

**Keywords:** gastric cancer, HNRNPA1, miR‐339, RP11‐81H3.2, tumor metastasis

## Abstract

Recent studies have demonstrated that various long non‐coding RNAs (lncRNAs) participate in the gastric cancer (GC) development and metastasis. Some lncRNAs exert their regulatory function by interacting with microRNAs. Here we identified a novel lncRNA RP11‐81H3.2 that was highly expressed in the GC tissue and cell lines. RP11‐81H3.2 knockdown significantly inhibited the proliferation, migration, and invasion of GC cells. Mechanistically, we demonstrated that RP11‐81H3.2 directly interacted with miR‐339 while miR‐339 regulated the HNRNPA1 expression by targeting HRRNPA1 3’‐UTR. RP11‐81H3.2‐miR‐339‐HNRNPA1 interaction network regulated the GC cell proliferation, migration, and invasion. Moreover, our results confirmed that RP11‐81H3.2 knockdown suppressed the tumor growth of GC in a xenograft model in vivo. In summary, the results suggest that RP11‐81H3.2 functions as an oncogene in GC and could be utilized as a promising diagnosis and therapeutic marker for GC treatment.

## Graphical abstract

LncRNA RP11‐81H3.2 is highly expressed in gastric cancer tissues and cell lines. Knockdown of lncRNA RP11‐81H3.2 suppresses gastric tumor growth in a xenograft tumor model. RP11‐81H3.2/miR‐339/HNRNPA1 interaction network regulates gastric cancer development and progession.

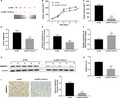

## 1 INTRODUCTION

Gastric cancer (GC) is one of the most common digestive tumors and ranked 2nd most common cause of death in the world.^1^, ^2^ Though the overall survival rate of GC has been improved substantially in the past few years due to the advances of the diagnostic technologies and therapeutic strategies, it remains difficult to cure as GC patients are usually diagnosed at a relative advanced stage.^3^ Thus, it is critical to identify the novel diagnostic markers and therapeutic targets in GC.

Long non‐coding RNAs (lncRNAs) are linear transcripts longer than 200 nucleotides without translating into proteins.^4^ LncRNAs have been reported to be involved in multiple biological processes and in various tumor development.^5^, ^6^, ^7^ Lots of lncRNAs participates in the GC development with different mechanisms, regulating cell proliferation, migration, invasion apoptosis, and tumor metastasis.^8^ LncRNA GHET1 exerts its regulatory functions by increasing c‐Myc mRNA stability and promoting the GC cell proliferation.^9^ Liu et al, reported that lncRNA HOTAIR regulated the GC development as a competing endogenous RNA (ceRNA) to sponge miR‐331‐3p and regulate the HER2 expression.^10^ Another group of lncRNA such as ANRIL, H19, and MEG3 exhibits their regulatory role by interacting with microRNAs (miRNAs).^11^, ^12^, ^13^

MicroRNAs are a group of small non‐coding RNAs which post‐transcriptionally regulate the gene expressions in tumor diagnosis, prognosis, and therapy.^14^ In gastric cancer, miRNAs have been reported to play critical roles in regulating expressions of known oncogenes or tumor suppressor genes.^15^ MiR‐339 was reported to exhibit a regulatory role in colorectal cancer,^16^ non‐small cell lung cancer,^17^ and breast cancer.^18^ Shen et al, found that miR‐339 could suppress the GC cell proliferation, migration, invasion, and tumor development via targeting NOVA1.^19^ Thus, miRNAs could be utilized as promising biomarkers for GC diagnosis and therapy.

Despite these progresses, our understanding about the lncRNA‐miRNA interaction network involved in the GC development is still unclear. Through high throughput nascent RNA capture sequencing, previous study showed that lncRNA RP11‐81H3.2 was highly expressed in multiple cancers, including stomach adenocarcinoma, colon adenocarcinoma, liver hepatocellular carcinoma, lung adenocarcinoma, lung squamous cell carcinoma etc, indicating its critical role in tumorigenesis.^20^

In the current study, we explored the role of lncRNA RP11‐81H3.2 in GC and found that knockdown RP11‐81H3.2 significantly inhibited the GC cell proliferation, migration, and invasion. Mechanistically, we defined the RP11‐81H3.2‐miR‐339‐HNRNPA1 interaction network regulating the GC development. In addition, using in vivo xenograft GC tumor model, we demonstrated that RP11‐81H3.2 knockdown suppressed the GC tumor growth. In summary, we suggest that RP11‐81H3.2 promotes the GC progression through RP11‐81H3.2‐miR‐339‐HNRNPA1 interaction network, which might provide a novel diagnosis and therapeutic marker for GC treatment.

## 2 MATERIALS AND METHODS

### 2.1 Clinical specimen

In this study, we collected 40 pairs of GC tissues and the adjacent normal tissues from those patients underwent surgical treatment at our hospital. This study was approved by the Ethics Committee of The Second Affiliated Hospital of Xi'an Jiaotong University (2016287‐XJTU). All the tissues were stored in liquid nitrogen before RNA isolation.

### 2.2 Cell culture

Four different GC cell lines, SGC‐7901, BGC‐823, AGS, HGC‐27, and MGC‐803 were obtained from American Type Culture Collection (ATCC). The normal gastric epithelium cell line (GES‐1) was purchased from Cell bank of Chinese Academy of Sciences and maintained in our lab. SGC‐7901, BGC‐823, HGC‐27, and MGC‐803 were cultured in Dulbecco's modified Eagle medium (DMEM) supplemented with 10% fetal bovine serum (FBS) and 1% penicillin‐streptomycin. GES‐1 and AGS were cultured in RPMI 1640 with 10% FBS and antibiotics. Mycoplasma detection was negative in all cell lines.

### 2.3 Transfection

Briefly, cells were seeded in 6‐well plates or 96‐well plates. When the confluence was approximately 60%‐70%, the medium was changed to serum‐free DMEM and the transfection was performed using Lipofectamine^TM^ 3000 (Invitrogen) according to manufacturer's protocols. Cells were generally assigned to different groups as follows: (a)negative control (NC) group and sh‐RP11‐81H3.2 group; (b) NC group and miR‐339 mimics group; (c) NC group, sh‐RP11‐81H3.2 group, miR‐339 mimics group and sh‐HNRNPA1 group. Cells were harvested at indicated time points for further experiments. The shRNA sequences used were as follows:

- siRNA1:GGTGTCAGAGAAGGCTGAATTGGGT
- siRNA2:CAGAGAAGGCTGAATTGGGTACCAA

- siRNA3:GAGAAGGCTGAATTGGGTACCAAGA

### 2.4 RT‐qPCR

Total RNA in tissues and cell lines was isolated using TRIzol Reagent (Invitrogen). cDNA was obtained through reverse transcription using PrimeScript RT reagent Kit (TaKaRa). Then real‐time PCR assay was performed by SYBR PrimeScript^TM^ PLUS RT‐PCR Kit (TaKaRa) to detect the expression level of RP11‐81H3.2, miR‐339, and HNRNPA1. The reaction condition of PCR was 95°C for 30 seconds, 60°C for 40 seconds and for 40 cycles. ACTB or U6 were used as an endogenous control for normalization. The relative expression levels were calculated by 2^−ΔΔCt^ methods. The primer sequences used were as follows: RP11‐81H3.2: forward, CCGGATGCCAGTCTACTACG, reverse, 5′‐TGATGTGCCAGGGAAGAAAGCCTA‐3′; miR‐339: forward, 5′‐TGCCAGTTAGTAGCCCAGAAGCAA‐3′, reverse, 5′‐TGATGTGCCAGGGAAGAAAGCCTA‐3′; HNRNPA1: forward, 5′‐AAGCAATTTTGGAGGTGGTG‐3′, reverse, 5′‐ATAGCCACCTTGGTTTCGTG‐3′; ACTB: forward, 5′‐TGTCACCAACTGGGACGATA‐3′, reverse, 5′‐GGGGTGTTGAAGGTCTCAAA‐3′; U6: forward, 5′‐ATTGGAACGATACAGAGAAGATT‐3′, reverse, 5′‐GGAACGCTTCACGAATTTG‐3′.

### 2.5 Cell proliferation assay

Cells were seeded in 96‐well plates with 2 × 10^3^ per well. After transfection, cell proliferation ability was evaluated by CCK‐8 assay (Dojindo). Cells were then cultured for 0, 24, 48, or 96 hours, after that, 10 μL of CCK‐8 (5 mg/mL) was added to the culture medium in each well. The absorbance at 450 nm was measured by Exl 800 microplate reader (Bio‐tek). All assays were independently performed in triplicate.

### 2.6 Cell apoptosis assay

After transfection, cells were cultured for 48 hours, and trypsinized to harvest. Then cells were fixed with 70% methanol and stained with FITC‐Annexin V and PI based on the instruction of the FITC Annexin V Apoptosis Detection Kit (BD Biosciences). The cells were analyzed with a flow cytometry (FACScan, BD Biosciences) equipped with a Cell Quest 3.0 software.

### 2.7 Wound healing assay

After transfection, 1 × 10^5^ SGC‐7901 or BGC‐823 cells were seeded in 6‐well plates and cultured until confluent. The wound is stimulated by scratching with a sterile 200 μL pipette tip. The floating cells were washed away and the remaining cells were cultured with serum‐free DMEM medium. An inverted optical microscope (Olympus) was used to monitor the closure of the wound at 0 and 48 hours.

### 2.8 Cell migration assay

The 24‐well transwell chambers (Costar) with Matrigel‐coated membranes were used for invasion assay. The cell concentration was adjusted to 1 × 10^5^ cells/mL. A total of 200 μL of the cell suspension was added into the upper chambers, while the bottom chambers were covered with 500 μL of DMEM supplemented with 10% of FBS. After 48h, the invading cells in the bottom chamber were stained with 0.1% crystal violet. The cells were observed and calculated under the inverted microscope (Olympus).

### 2.9 Western blotting

Cells were lysed in RIPA buffer with 1mM PMSF. The concentration of the total protein obtained was quantified using a BCA protein assay kit (Thermo). Proteins were separated on 10% SDS‐PAGE and electro‐transferred to the PVDF membrane (Millipore). And then were incubated with anti‐HNRNPA1 (Abcam) and anti‐β‐actin (Abcam) at 4°C overnight, then incubated with an HRP‐labelled secondary antibody IgG (Abcam) at room temperature for 1h. Immunolabeling was visualized using the ECL system (Millipore).

### 2.10 Luciferase reporter assay

The 3’‐UTR of RP11‐81H3.2 containing the predicted miR‐339 binding site was amplified by PCR. And then was cloned into a Dual‐luciferase miRNA Target Expression Vector (Promega) to construct RP11‐81H3.2‐wild type (RP11‐81H3.2‐Wt). The same approach was used to construct RP11‐81H3.2‐mutated type (RP11‐81H3.2‐Mut). Similarly, HNRNPA1‐wild type (HNRNPA1‐Wt) and HNRNPA1‐mutated type (HNRNPA1‐Mut) were set up. Then the luciferase activities were tested by Dual‐luciferase reporter assay system (Promega).

### 2.11 Tumor xenograft model

The posterior flank of the 6‐week‐old male BALB/c nude mice (n = 12) were subcutaneously injected with SGC‐7901 (2 × 10^7^) cells transfected with sh‐HNRNPA1 or negative control. Tumor volumes were examined every 4 days, and tumor tissues were photographed and weighed on Day 17. The expression level of miR‐339 in tumor tissues was detected by qRT‐PCR. The protein level of HNRNPA1 in tumor tissues was measured by western blot. The animal experiment was performed in compliance with the authenticated animal protocols of Ethical Committee of Animal Welfare of our hospital.

### 2.12 Statistical analysis

Statistical analyses were performed with GraphPad Prism 6.0 software and data were expressed as mean ± SD. Statistical comparisons were made by one‐way analysis of variance (ANOVA) or student t test. *P* < .05 indicated a statistically significant difference.

## 3 RESULTS

### 3.1 RP11‐81H3.2 is highly expressed in GC tissues and cell lines

**Figure 1:**
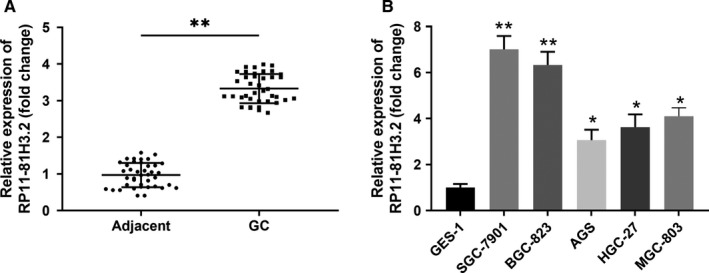
RP11‐81H3.2 is highly expressed in GC tissues and cell lines. A, The relative expression of lncRNA RP11‐81H3.2 in GC tissues and adjacent normal tissues; (B) The relative expression of lncRNA RP11‐81H3.2 in human GC cell lines (SGC‐7901, BGC‐823, AGS, HGC‐27, and MGC‐803) and normal GES‐1. LncRNA RP11‐81H3.2 expression was analyzed by qPCR and normalized to the expression of ACTB. *P < .05 compared with GES‐1; **P＜.01 compared with adjcent normal tissues or GES‐1

To explore the role of RP11‐81H3.2 in GC, we first examined the expression levels of RP11‐81H3.2 in GC tissues and cell lines. We found that the expression of RP11‐81H3.2 was significantly higher in GC tissues compared with that in the adjacent normal tissues (Figure 1A). In addition, the expression of RP11‐81H3.2 was also remarkably higher in three different GC cell lines (SGC‐7901, BGC‐823, AGS,HGC‐27, and MGC‐803) (Figure 1B), which indicated RP11‐81H3.2 might be involved in the GC progression. Since SGC‐7901 and BGC‐823 had a relatively higher RP11‐81H3.2 expression levels, these two cell lines were used for the subsequent experiments.

Moreover, to assess the clinical significance of RP11‐81H3.2 expression in gastric patients, we analyzed the association of RP11‐81H3.2 expression with the clinical features from 40 gastric patients. Based on the individual RP11‐81H3.2 expression level, patient samples were grouped into either a high‐ or low‐expressing group. We discovered that expression level of RP11‐81H3.2 was positively correlated to TNM stage, metastasis, (Table 1).

**Table 1:** Association between RP11‐81H3.2 and the clinical features

Characteristic	RP11‐81H3.2 expression		*P* value
	Low (n = 20)	High (20)	
Age (y)			
≤65	8	10	.525
＞65	12	10	
Gender			
Male	16	14	.465
Female	4	6	
TNM stage			
I + II	14	7	.0267
III + IV	6	13	
Lymph node metastasis			
Negative	9	3	.0384
Positive	11	17	

### 3.2 RP11‐81H3.2 knockdown inhibits the proliferation, migration, and invasion of GC cells

**Figure 2:**
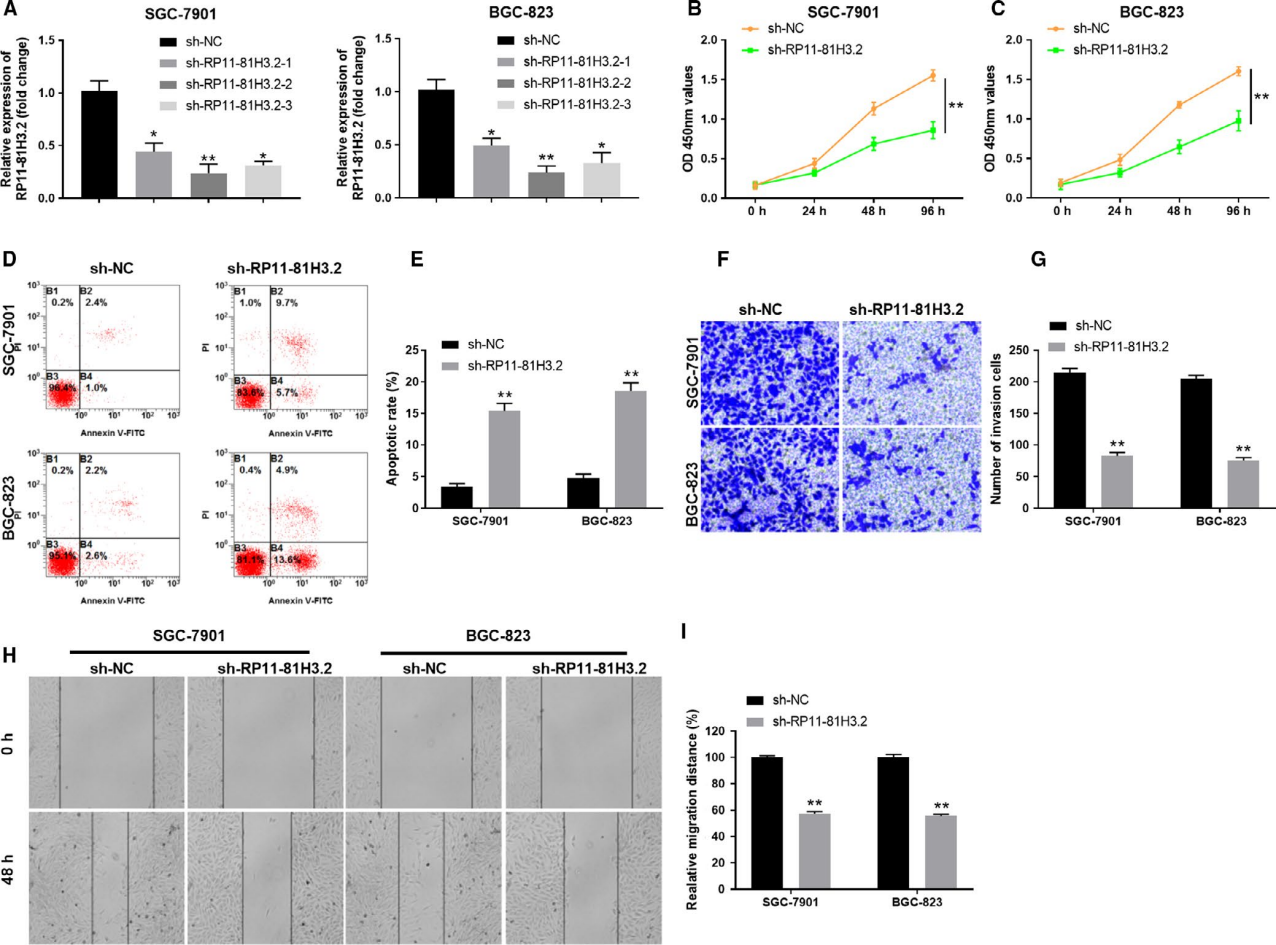
RP11‐81H3.2 knockdown inhibits the proliferation, migration, and invasion of GC cells. A, SGC‐790 1 and BGC‐823 cells were transfected with RP11‐81H3.2 knockdown vector (sh‐RP11‐81H3.2) or negative control vector (sh‐NC). The knockdown efficiency was examined by qPCR. B, C, The cell proliferation of SGC‐7901 and BGC‐823 cells transfected with sh‐RP11‐81H3.2 or sh‐NC was assessed by CCK‐8 kit at indicated time points. D‐E, The apoptosis of cells with knockdown RP11‐909N7.2 was determined by the flow cytometry. F‐G, The cell invasion capability of SGC‐7901 and BGC‐823 cells transfected with sh‐RP11‐81H3.2 or sh‐NC was analyzed by transwell assay. (H‐I) Cell migration capability of SGC‐7901 and BGC‐823 cells transfected with sh‐RP11‐81H3.2 or sh‐NC was analyzed by wound healing assay.*P < .05, **P < .01, compared with sh‐NC group

To investigate the function of RP11‐81H3.2, we utilized the shRNA knockdown to silence the expression of RP11‐81H3.2. As shown in Figure 2A, SGC‐7901 and BGC‐823 GC cells transfected with knockdown vector (sh‐RP11‐81H3.2) significantly downregulated the RP11‐81H3.2 expression. The most efficient shRP11‐81H3.2‐3 was used for the subsequent experiments (Figure 2A). CCK‐8 assay showed that the cell proliferation in SGC‐7901 and BGC‐823 GC cells were both remarkably inhibited after sh‐RP11‐81H3.2 transfection for 96 hours compared with that in cells transfected with sh‐NC control (Figure 2B,C). In addition, knockdown of RP11‐81H3.2 significantly enhanced GC cell apoptosis (Figure 2D,E). Moreover, transwell invasion assay and wound healing assay were carried out and the results demonstrated that silencing RP11‐81H3.2 remarkably suppressed cell invasion (Figure 2F,G) and inhibited the relative migration distance of SGC‐7901 and BGC‐823 cells in the scratch wounds (Figure 2H,I). To exclude the off‐target effect of RP11‐81H3.2 knockdown, we used another shRNA (shRP11‐81H3.2‐3) and obtained the similar effect of RP11‐81H3.2 knockdown on cell proliferation and invasion (Figure S1).

### 3.3 RP11‐81H3.2 directly interacts with miR‐339

**Figure 3:**
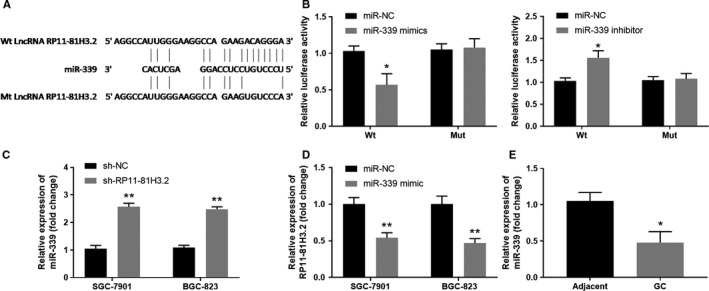
RP11‐81H3.2 directly interacts with miR‐339. A, Bioinformatics analysis predicted the potential RP11‐81H3.2 binding sites of miR‐339. B, HEK293 cells were transfected luciferase reporter vector containing lncRNA RP11‐81H3.2 WT or mutant sequences, together with miR‐NC control, miR‐339 mimics, or miR‐339 inhibitor. Relative luciferase activity was examined using a due‐luciferase reporter kit. C, SGC‐7901 and BGC‐823 GC cells were transfected with sh‐NC or sh‐RP11‐8H3.2, miR‐339 expression was analyzed by qPCR. D, SGC‐7901 and BGC‐823 GC cells were transfected with miR‐NC or miR‐339 mimics, lncRNA RP11‐81H3.2 expression was analyzed by qPCR. E, The expression levels of miR‐339 in GC tissues and adjacent normal tissues were analyzed by qPCR. *P < .05, compared with miR‐NC or adjacent normal tissues; **P＜.01, compared with sh‐NC or miR‐NC

Long non‐coding RNAs (LncRNAs) could exert their functions as competing endogenous RNAs (ceRNAs) by interaction with miRNAs in regulating target gene mRNA levels.^21^ We searched for the RP11‐81H3.2 targets using bioinformatics database Lncbase and identified miR‐339 could be a potential miRNA target binding by RP11‐81H3.2 (Figure 3A). Luciferase reporter assay demonstrated that RP11‐81H3.2 directly interacted with miR‐339 as overexpression miR‐339 could significantly inhibit the luciferase activity of reporter containing RP11‐81H3.2 WT sequence, but not the reporter containing mutant sequence (Figure 3B). However, miR‐339 inhibitor enhanced the relative luciferase activity in HEK293 cells transfected with reporter containing RP11‐81H3.2 WT sequence (Figure 3B). Next, we further tested the regulation between RP11‐81H3.2 and miR‐339. As shown in Figure 3C,D, knockdown RP11‐81H3.2 using sh‐RP11‐81H3.2 notably upregulated the miR‐339 expression in GC cells while overexpression miR‐339 using miR‐339 mimics dramatically decreased the RP11‐81H3.2 expression levels. Intriguingly, we also detected significantly lower levels of miR‐339 in GC tissues compared with those in the adjacent normal tissues (Figure 3E).

### 3.4 Overexpression of miR‐339 inhibits the proliferation, migration and invasion of GC cells, and enhances GC cell apoptosis

**Figure 4:**
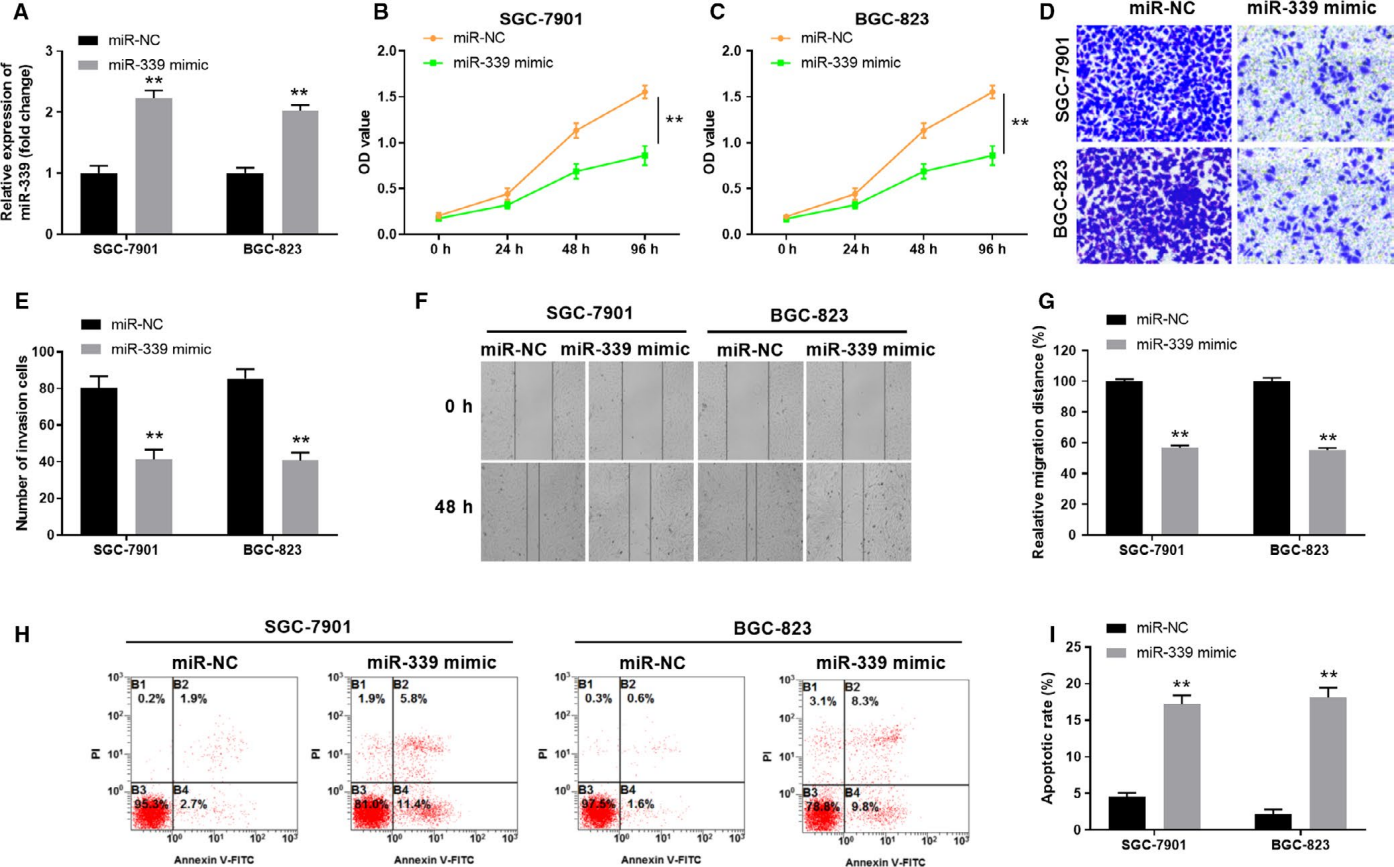
Overexpression of miR‐339 inhibits the proliferation, migration, and invasion of GC cells, and enhances GC cell apoptosis. A, SGC‐7901 and BGC‐823 cells were transfected with miR‐339 mimic or negative control vector (miR‐NC). The relative expression of miR‐339 was examined by qPCR. B, C, The cell proliferation of SGC‐7901 and BGC‐823 cells transfected with miR‐NC or miR‐339 mimic was assessed by MTT assay at indicated time points. D, E, The cell invasion capability of SGC‐7901 or BGC‐823 cells transfected with miR‐NC or miR‐339 mimic was analyzed by transwell assay. F, G, The cell migration capability of SGC‐7901 and BGC‐823 cells transfected with miR‐NC or miR‐339 mimic was analyzed by wound healing assay. H, I, The apoptosis of GC cells were determined by the flow cytometry. ** *P* < .01 compared with miR‐NC group

To further explore the function of miR‐339 on GC cells, we overexpressed miR‐339 in SGC‐7901 or BGC‐823 cells by using miR‐339 mimic (Figure 4A). As shown in Figure 4B,C, MTT assay showed that the cell proliferation in SGC‐7901 and BGC‐823 GC cells were both remarkably inhibited 96 hours post miR‐339 mimic transfection compared with that in cells transfected with sh‐NC control. In addition, transwell invasion assay and wound healing assay were carried out and the results demonstrated that overexpression of miR‐339 remarkably suppressed cell invasion and migration, respectively (Figure 4D,G) Furthermore, our data showed that overexpression of miR‐339 enhanced the GC cell apoptosis (Figure 4H,I). Together, the results suggest that overexpression of miR‐339 inhibits the proliferation, migration and invasion of GC cells, and enhances GC cell apoptosis.

### 3.5 miR‐339 regulates HNRNPA1 expression by targeting HNRNPA1 3’‐UTR

**Figure 5:**
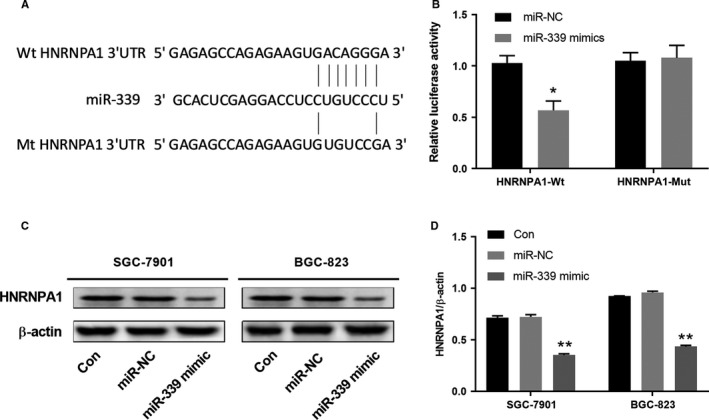
miR‐339 regulates the HNRNPA1 expression by targeting HNRNPA1 3’‐UTR. A, Bioinformatics analysis indicted the putative complementary binding sites between HNRNPA1 and miR‐339. B, HEK293 cells were co‐transfected with miR‐339 mimics or miR‐NC control and luciferase reporter vectors containing WT 3’‐UTR of HNRNPA1 or mutated 3’‐UTR of HNRNPA1. Relative luciferase activity was analyzed 48 h later. C‐D, GC cell line SGC‐7901 or BGC‐823 was transfected with miR‐NC, miR‐339 mimics or left untreated. HNRNPA1 protein expression was analyzed 48 h later. The representative western blot data were shown and the experiments were repeated at least three times independently. *P < .05, compared with miR‐NC; **P ＜.01, compared with the control

miR‐339 was reported regulating the p53 tumor‐suppressor pathway in cancer development.^22^ Searching miR‐339 target genes using different bioinformatics software (TargetScan, miRDB, and DIANA‐Tool) indicated that HNPNPA1 as a potential target (Figure 5A). To further validate the prediction, we constructed luciferase reporter vectors containing WT 3’‐UTR of HNRNPA1 or mutated 3’‐UTR of HNRNPA1 and tested whether the luciferase activity could be regulated by miR‐339.The results showed that relative luciferase activities in HEK293 cells transfected with the reporter vector containing the WT HNRNPA‐1 3’‐UTR, but not the mutant sequences could be inhibited by co‐transfecting with the miR‐339 mimics (Figure 5B). We further tested the HNRNPA1 protein expression levels in GC cells after miR‐339 mimics transfection. miR‐339 overexpression in SCC‐7901 and BGC‐823 cells indeed suppressed the expression of HNRNPA1 (Figure 5C,D). Taken together, miR‐339 directly targets the 3’‐UTR of HNRNPA1 to suppress the expression of HNRNPA1 protein.

### 3.6 RP11‐81H3.2‐miR‐339‐HNRNPA1 interaction network regulates the GC cell proliferation, migration, and invasion

**Figure 6:**
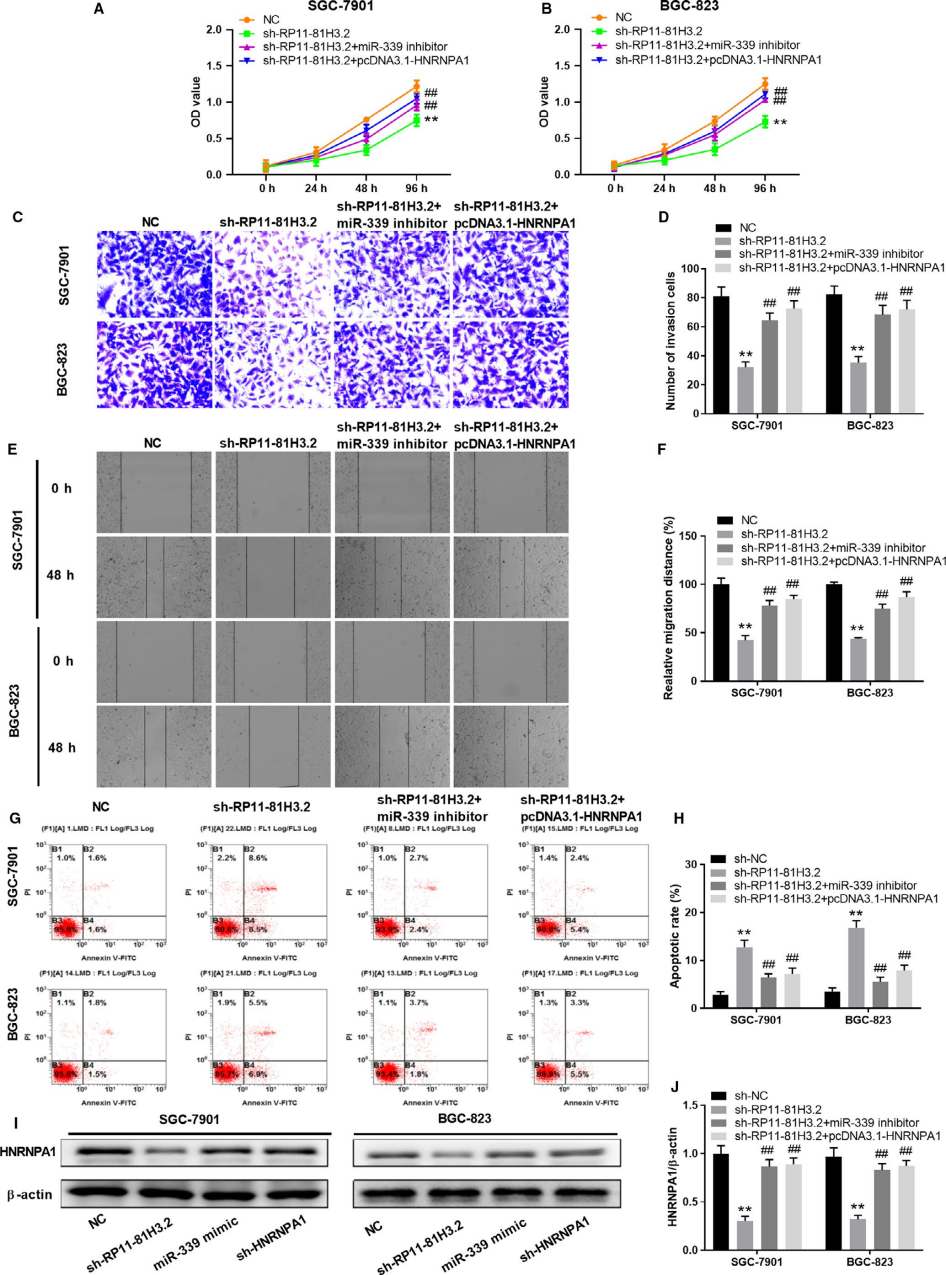
RP11‐81H3.2‐miR‐339‐HNRNPA1 interaction network regulates the GC cell proliferation, migration, and invasion. SGC‐7901 or BGC‐823 cells were transfected with RP11‐81H3.2 knockdown vector (sh‐RP11‐81H3.2), sh‐RP11‐81H3.2 + miR‐339 inhibitor, sh‐RP11‐81H3.2 + pcDNA3.1‐HNRNPA1, or NC. A, B, The cell proliferation of SGC‐7901 or BGC‐823 cells was assessed by CCK‐8 assay at indicated time points. C, D, The cell invasion capability of SGC‐7901 and BGC‐823 cells was analyzed by transwell assay. E, F, The cell migration capability of SGC‐7901 and BGC‐823 was analyzed by wound healing assay. G, H, cells were stained with Annexin V/PI and cell apoptosis was analyzed by flow cytometry; I, J, HNRNPA1 protein expression was analyzed 48 h later. The representative western blot data were shown and the experiments were repeated at least three times independently. **P < .01 vs NC group, ^##^ *P* < .01 vs sh‐RP11‐81H3.2 group

To further investigate the function of RP11‐81H3.2‐miR‐339‐HNRNPA1 interaction network, we transfected SGC‐7901 and BGC‐823 GC cells with sh‐RP11‐81H3.2, miR‐339 mimics, sh‐HNRNPA1, or negative control. As shown in Figure 6A,B, compared with NC control, SGC‐7901 and BGC‐823 GC cells transfected with sh‐RP11‐81H3.2, miR‐339 mimics, or sh‐HNRNPA1 significantly inhibited the cell proliferation as examined by CCK‐8 kit. In addition, transwell invasion assay and wound healing assay were carried out and the results demonstrated that silencing RP11‐81H3.2 or HNRNPA1, or overexpression miR‐339 remarkably suppressed cell invasion, respectively (Figure 6C,D) and drastically inhibited the relative migration distance of SGC‐7901 and BGC‐823 cells in the scratch wounds (Figure 6E,F).Reversely, SGC‐7901 and BGC‐823 GC cells transfected with sh‐RP11‐81H3.2, miR‐339 mimics, or sh‐HNRNPA1exhibited remarkably higher cell apoptosis (Figure 6G,H).We further tested the HNRNPA1 protein expression levels in GC cells with different treatments. Compared with NC group, RP11‐81H3.2 knockdown and miR‐339 overexpression significantly inhibited the protein expression of HNRNPA1, while HNRNPA1 knockdown group showed the lowest levels of HNRNPA1 protein (Figure 6I,J).

### 3.7 RP11‐81H3.2 knockdown suppresses tumor growth of GC in a xenograft model

**Figure 7:**
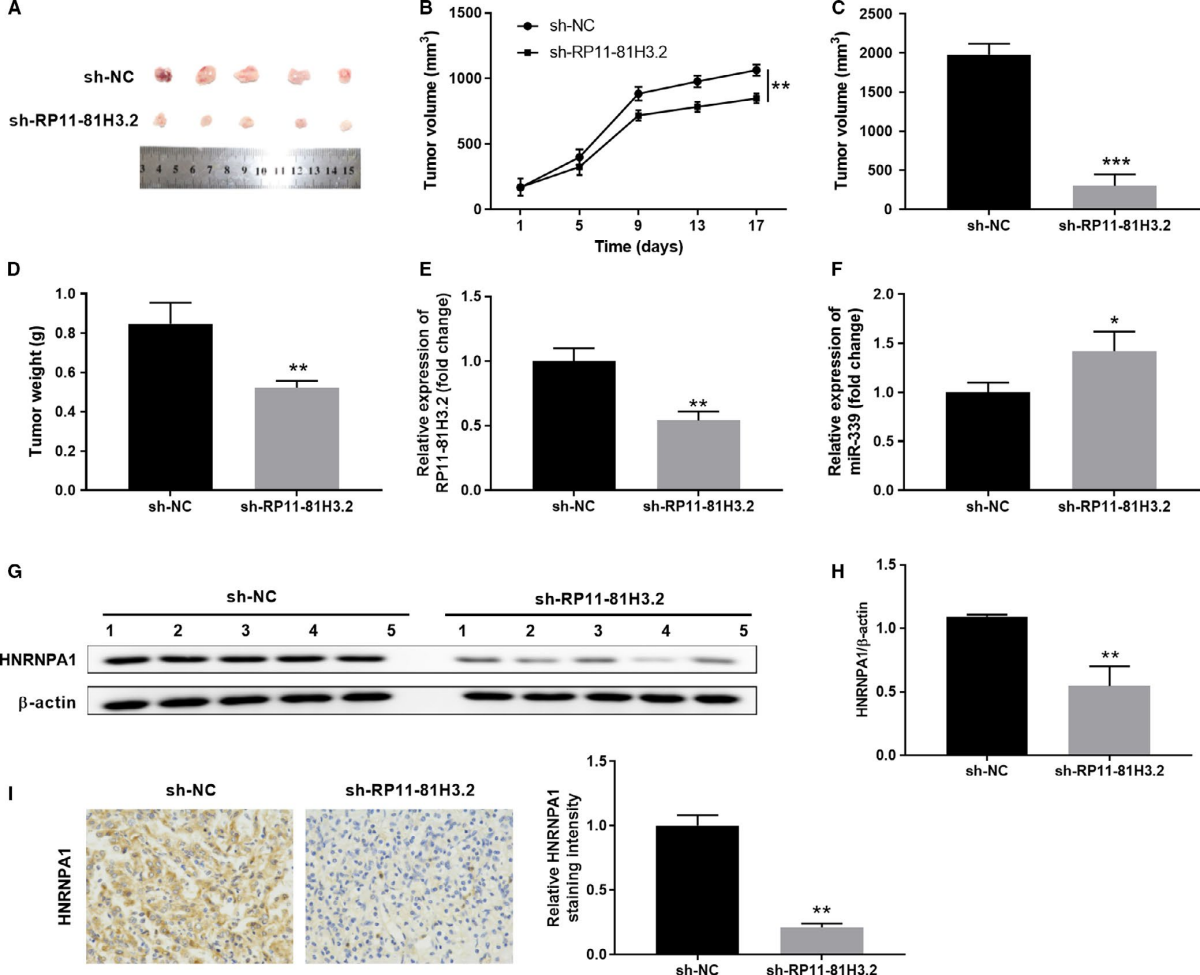
RP11‐81H3.2 knockdown suppresses the tumor growth of GC in a xenograft model. A, Photographs of tumor tissues from sh‐NC or sh‐RP11‐81H3.2 group were taken at Day 17. B‐D, Tumor growth curves in xenografts of nude mice and tumor volume/weight were examined. E, F, The relative expression of RP11‐81H3.2 and miR‐339 was analyzed by qPCR. (G, H) HNRNPA1 protein expression levels in tumor tissues of different groups were determined by western blot. (I) Immunohistochemical staining of HNRNPA1 in tumor tissues of different groups were performed. *P < .05, **P < .01, ***P < .001, compared with sh‐NC

In vitro results indicated that RP11‐81H3.2 could inhibit the GC cell proliferation and metastasis. Thus, we further examined whether RP11‐81H3.2 affected GC tumor development in vivo. SGC‐7901 cells were stably transfected with negative control (sh‐NC) or sh‐RP11‐81H3.2 and implanted subcutaneously into nude mice to develop tumor. Tumor sizes were examined every 4 days after implantation and mice were euthanized at Day 17 (Figure 7A). Compared with sh‐NC group, the xenograft tumor size was drastically smaller in sh‐RP11‐81H3.2 group (Figure 7B,C) and tumor weight was decreased (Figure 7D). We also checked the RP11‐81H3.2 and HNRNPA1 expression in tumor tissues. As shown in Figure 7E,F, tumor tissues from sh‐RP11‐81H3.2 group showed significantly lower level of RP11‐81H3.2 and higher level of miR‐339 expression.

Moreover, the HNRNPA1 expression was downregulated compared with those in tumor tissues from sh‐NC group (Figure 7G,I). Overall, our results indicate that RP11‐81H3.2 knockdown suppresses the tumor growth of GC in a xenograft model.

## 4 DISCUSSION

Mounting evidences suggest that lncRNAs participate in different stages of GC development and progression, which could be utilized for diagnosis and treatment of GC.^8^ Previous study showed that lncRNA RP11‐81H3.2 was highly expressed in multiple cancers as demonstrated by high throughput nascent RNA capture sequencing.^20^ In this study, we further evaluated the expression pattern and function role of RP11‐81H3.2 in GC. Moreover, we have identified the RP11‐81H3.2‐miR‐339‐HNRNPA1 regulatory axis in GC development and metastasis.

There are few studies about the function of RP11‐81H3.2. We confirmed that RP11‐81H3.2 played an “oncogenic” role in GC development and knockdown RP11‐81H3.2 expression inhibited the GC cell proliferation, migration, and invasion. RP11‐81H3.2 exerts its function as a ceRNA via sponging miR‐339. Haijun Li et al reported that reduced miR‐339 expression predicts the poor prognosis of GC. In ovarian cancer, miR‐339 inhibited the tumor progress by directly regulating NACC1 and Bcl6 while miR‐339 regulated the metastasis of colorectal cancer via targeting PRL‐1.^23^, ^24^ LncRNA RP11‐81H3.2 might function as a ceRNA and sponge the miR‐339. How silencing of RP11‐81H3.2 increases the expression of miR‐339 was not fully understood in the study. However, lncRNA might regulate the expression and function of miRNA via sponging or decoying the miRNA. LncRNA might also regulate the expression of miRNA by controlling the maturation process of miRNA. For instance, lncRNA CCAT2 that is mainly located in the nucleus selectively blocks miR‐145 maturation by inhibiting pre‐miR‐145 export to cytoplasm.^25^ Here RP11‐81H3.2 might have the same mechanism regulating the expression of miR‐339. However, how miR‐339 functions in GC has not been studied. We identified that miR‐339 regulated the HNRNPA1 expression by targeting HNRNPA1 3’‐UTR.

HNRNPA1 is an important protein functions in RNA metabolism and involved in the cancer development and metastasis.^26^, ^27^ High level of HNRNPA1 indicates poor prognosis of hepatocellular carcinoma and HNRNPA1 overexpression promotes tumor metastasis.^28^ In ovarian cancer, published results showed that miR‐15a‐5p and miR‐25‐3p negatively regulated HNRNPA1 expression level.^29^ A previous study demonstrated that HNRNPA1 promoted cell invasion in GC by inducing epithelial‐to‐mesenchymal transition.^30^ In the current study, we revealed that knockdown HNRNPA1 could also inhibit cell proliferation, migration, and invasion while promote cell apoptosis in GC cell lines. Intriguingly, inhibition of miR‐339 or overexpression of HNRNPA1 abrogated the regulatory effect of RP11‐81H3.2 knockdown, which indicates the interaction axis among RP11‐81H3.2‐miR‐339‐HNRNPA1.

It is interesting to note that this is the first report to define the function of RP11‐81H3.2 in GC. Whether it exhibits similar oncogenic function in other tumors needs further investigation. In addition, due to the multiple‐to‐multiple relationships between miRNAs and target genes, we could not exclude the possibility that miR‐339 might inhibited GC development and metastasis via multiple mechanisms.^31^ Taken together, we demonstrated that RP11‐81H3.2 could promote the GC progression through RP11‐81H3.2‐miR339‐HNRNPA1 interaction network, which provides a novel diagnosis and therapeutic marker for GC treatment.

## 5 CONCLUSION

In summary, we herein verified RP11‐81H3.2 as a biomarker of GC. Knockdown RP1‐81H3.2 inhibited the GC cell proliferation, migration, and invasion. Mechanistically, we have demonstrated that RP11‐81H3.2 directly interacted with miR‐339 and miR‐339 regulated the HNRNPA1 expression by targeting HNRNPA1 3’‐UTR. The results suggest that RP11‐81H3.2 functions as an oncogene in GC development and metastasis and could be utilized as a promising diagnosis and therapeutic marker for GC treatment.

## CONFLICT OF INTEREST

The authors declare that they have no conflict of interest.

## ETHICS APPROVAL AND CONSENT TO PARTICIPATE

This study was approved by the Ethics Committee of The Second Affiliated Hospital of Xi'an Jiaotong University.

## Data Availability

The datasets used and/or analyzed during the current study are available from the corresponding author on reasonable request.
