# Supplementary figures and images for: RP11‐81H3.2 promotes gastric cancer progression through miR‐339‐HNRNPA1 interaction network
Source: Cancer Med. 2020 Feb 13;9(7):2524–34. doi: 10.1002/cam4.2867 (PMC7131847; doi:10.1002/cam4.2867)

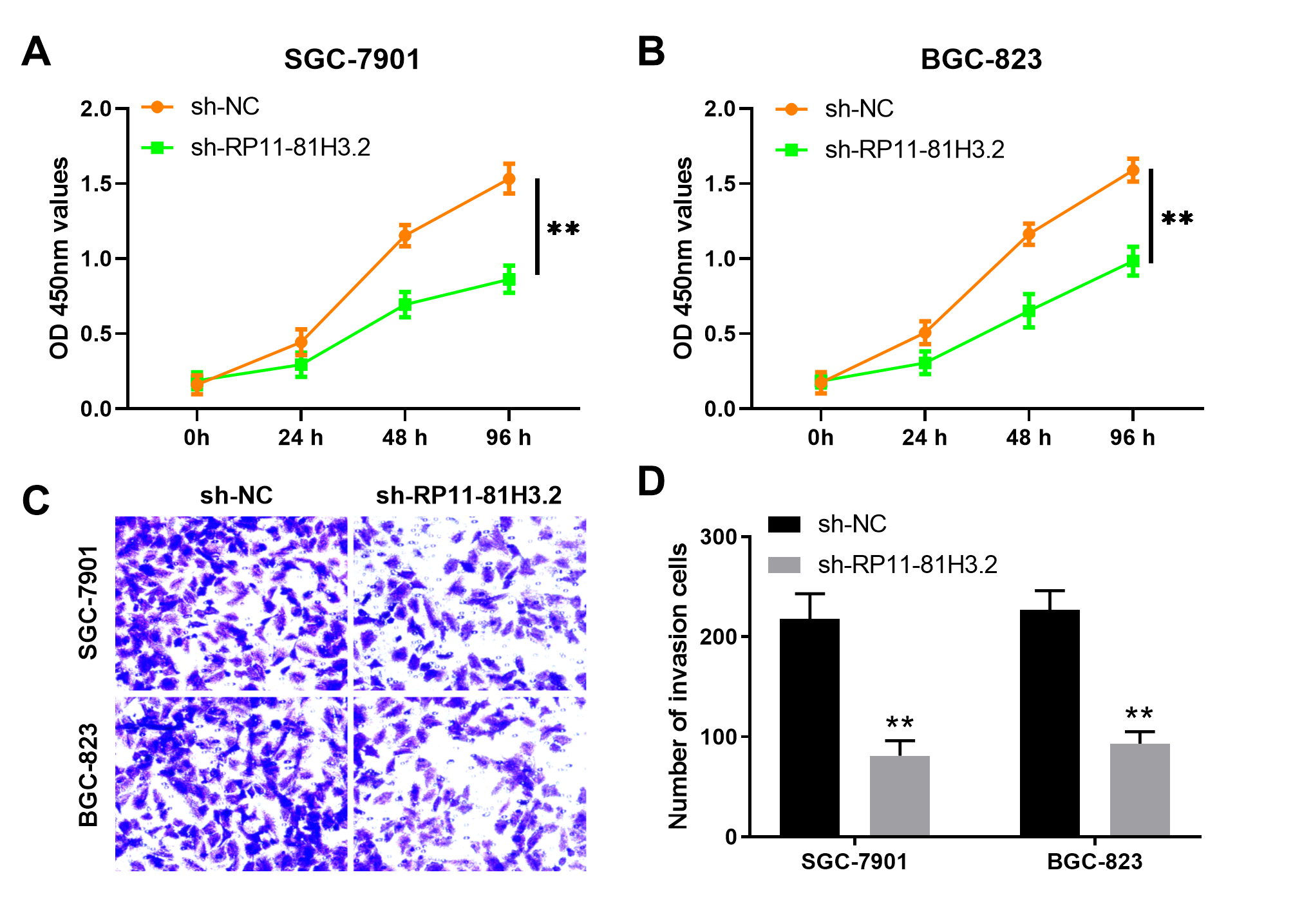

Supplement: Supplementary file 1 [file CAM4-9-2524-s001.tif]
